# High resolution 7T and 9.4T-MRI of human cerebral arterial casts enables accurate estimations of the cerebrovascular morphometry

**DOI:** 10.1038/s41598-018-32427-w

**Published:** 2018-09-24

**Authors:** Jasper H. G. Helthuis, Albert van der Zwan, Tristan P. C. van Doormaal, Ronald L. A. W. Bleys, Anita A. Harteveld, Annette van der Toorn, Mariana Brozici, Jeroen Hendrikse, Jaco J. M. Zwanenburg

**Affiliations:** 1 University Medical Center Utrecht, Brain Center Rudolf Magnus, Department of Neurology and Neurosurgery, Utrecht, The Netherlands; 2 University Medical Center Utrecht, Department of Anatomy, Utrecht, The Netherlands; 3 Brain Technology Institute, Utrecht, The Netherlands; 4 University Medical Center Utrecht, Department of Radiology, Utrecht, The Netherlands; 5 Heilig Hart Ziekenhuis, Department of Pulmonology, Mol, Belgium; 6 Biomedical MR Imaging and Spectroscopy Group, Center for Image Sciences, University Medical Center Utrecht, Utrecht, The Netherlands

## Abstract

Quantitative data on the morphology of the cerebral arterial tree could aid in modelling and understanding cerebrovascular diseases, but is scarce in the range between 200 micrometres and 1 mm diameter arteries. Traditional manual measurements are difficult and time consuming. 7T-MRI and 9.4T-MRI of human cerebral arterial plastic casts could proof feasible for acquiring detailed morphological data of the cerebral arterial tree in a time efficient method. One cast of the complete human cerebral arterial circulation embedded in gadolinium-containing gelatine gel was scanned at 7T-MRI (0.1 mm isotropic resolution). A small section of another cast was scanned at 9.4T-MRI (30 µm isotropic resolution). Subsequent 3D-reconstruction was performed using a semi-automatic approach. Validation of 7T-MRI was performed by comparing the radius calculated using MRI to manual measurements on the same cast. As manual measurement of the small section was not feasible, 9.4T-MRI was validated by scanning the small section both at 7T-MRI and 9.4T MRI and comparing the diameters of arterial segments. Linear regression slopes were 0.97 (R-squared 0.94) and 1.0 (R-squared 0.90) for 7T-MRI and 9.4T-MRI. This data shows that 7T-MRI and 9.4T-MRI and subsequent 3D reconstruction of plastic casts is feasible, and allows for characterization of human cerebral arterial tree morphology.

## Introduction

Quantitative data on the morphology of the cerebral arterial tree could lead to better understanding of cerebral blood flow and could aid in modelling and understanding cerebrovascular diseases^1^. One example would be generation of a general hemodynamic model for study on specific diseases, such as stroke. A secondary option is to generate boundary conditions in patient-specific aneurysm flow models, for planning and evaluating the risk of by-pass surgery. The cerebral arterial resistance is known to be generated largely by the arterioles and capillaries^2,3^.

However, to our knowledge only a limited number of studies to quantify this morphology of arteries in the range between 200 micrometres and 1 mm diameter have been performed^4–6^. The paucity of data in this range hampers the development of both general and patient-specific models of the entire cerebral arterial circulation.

The larger end of the 0.2–1 mm range can be obtained by *in vivo* angiographic imaging with MRI,3D computed tomography angiography (CTA) or 3D digital subtraction angiography^6,7^. However, even with modern techniques the smaller end is not well visualized *in vivo* and is vulnerable to measurement errors due to motion of the subject.

Detailed morphological data over the entire range could be acquired by manual measurements on corrosion casts of the cerebral arterial tree which are obtained by injecting a solidifying material (e.g. plastic) in the major cerebral arteries. These casts can retain arteries up to the pre-arteriole level, after removing brain tissue by corroding with substances such as potassium hydroxide. An alternative is manual dissection of arteries and subsequent measurement^8–10^. However, measurements on casts and dissection are difficult and time consuming to perform on smaller arteries. Micro-CT and confocal laser microscopy can ease these measurements. Availability of scanners which can handle larger samples at a high resolution are limited. 7T magnetic resonance imaging (MRI) has the advantage of reaching high resolutions combined with a larger field of view, comparable to industrial micro-CT systems. 7T MRI could proof to be a good alternative to micro-CT, especially when the latter is not available or when local available systems cannot handle large sample sizes^5,11–14^. Additionally, a (semi)-automatic subsequent 3D reconstruction technique could ease and greatly speed up the measurement process. This technique could fill the gap of missing data in the 0.2–1 mm range by scanning polymer cerebral arterial casts.

The aim of the current study was to access the feasibility of 7T-MRI and 9.4T-MRI scanning of human cerebral arterial plastic casts for acquiring detailed morphological data for a large part of the cerebral arterial tree.

## Methods

### Cast preparation

**Figure 1:**
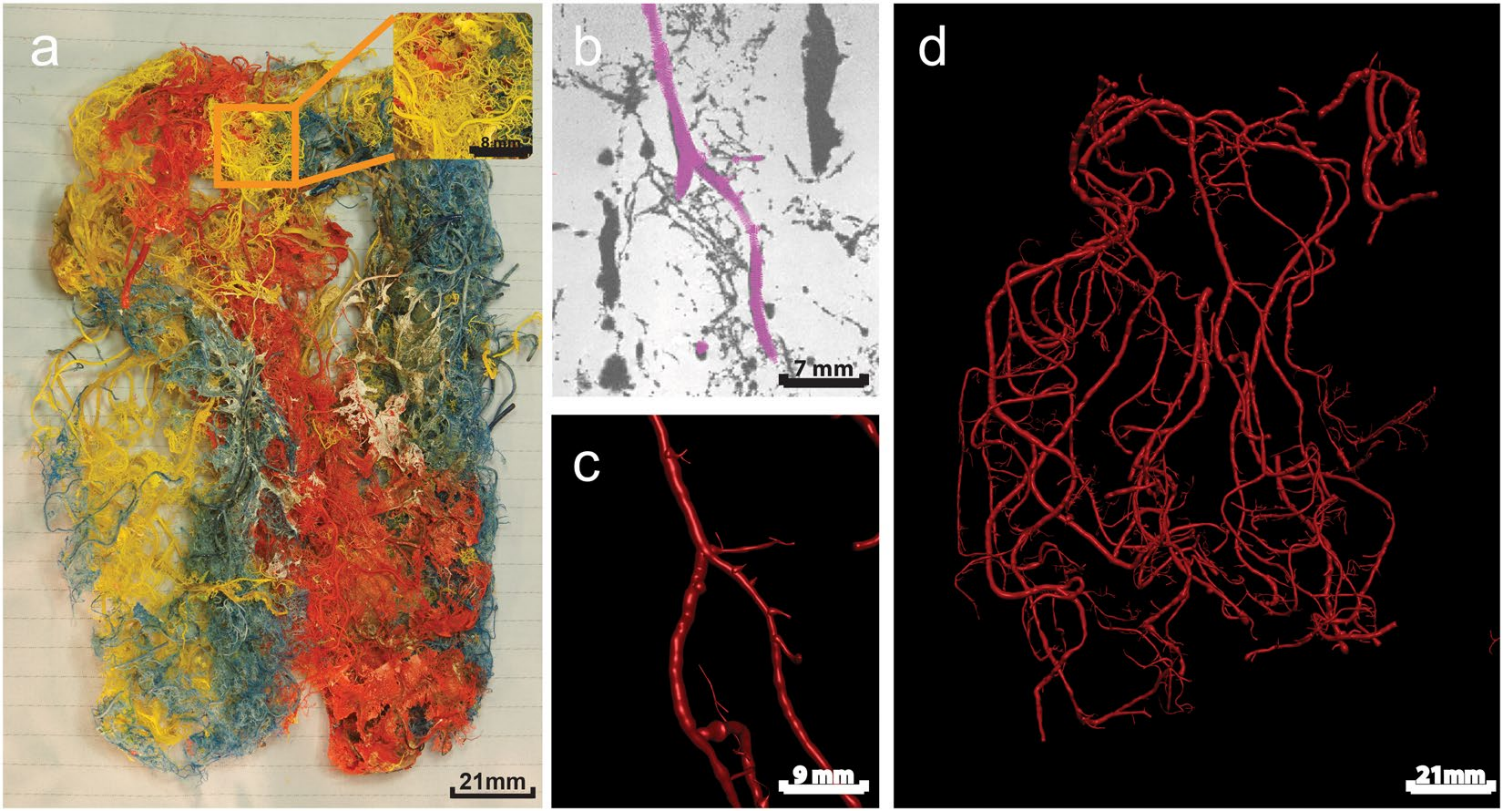
A human cerebral cast scanned with 7T MRI. (**a**) Photographic image of the complete cast. The inset shows the enlarged section indicated by the orange square. Different pigments where used for different major cerebral arteries during injection of Araldite F. (**b**) Detail of a 7T MRI image (100 µm isotropic resolution), with segmented artery as displayed in Simple Neurite Tracer. (**c**) The segmented artery of panel b in 3D representation. (**d**) 3D visualisation of all reconstructed arteries. As the reconstruction approach was semi-automatic not all arteries in the casts are segmented, only arteries which were manually selected in Simple Neurite Tracer are reconstructed to 3D.

**Figure 2:**
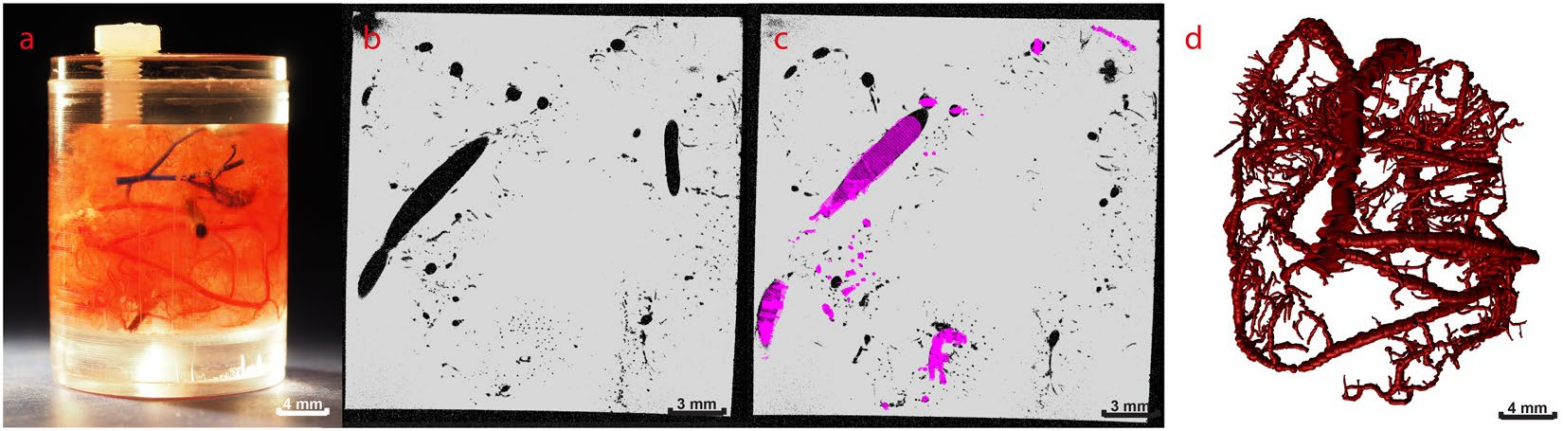
Small section of the human cerebral cast scanned with 9.4T MRI. (**a**) The cast section embedded in gel in a small plastic container, (**b**) Single slice from MRI (30 µm isotropic resolution), (**c**) Segmented arteries on single slice as displayed in Simple Neurite Tracer. (**d**) 3D visualisation of all reconstructed arteries.

Casts were available from a previous unpublished study in which casts were stored after air-drying. These casts were made from vessels of two brains that were derived from bodies that were donated to the Department of Anatomy. From these persons written informed consent was obtained during life that allowed the use of their entire bodies for educational and research purposes. In accordance with the Dutch law on dead bodies no IRB approval was required. There was no knowledge of cerebrovascular disease in the subjects and the cause of death was not related with any cerebrovascular disease. The unembalmed human brains (both females, 78 and 57 years) were obtained by cutting its connecting structures to the skull base after removal of the skull cap. Casts (see Figs 1a and 2a) were produced by cannulating the six major cerebral arteries of the brain after the circle of Willis and injecting these with a mixture of Araldite F/hardener HY 2967/dilutioner DY 026 SP and by subsequently corroding them, as described previously in more detail^15^.

### Preparation for scanning

#### 7T-MRI

A challenge for scanning polymer vessel casts with MRI is that the solid polymers do not yield an MRI detectable signal. This could be overcome by embedding the cast in a gadolinium-containing gel. The cast (female, 78 years) was placed in a 14% gelatine tap water gel. To increase the attainable signal-to-noise ratio per unit scan time for high resolution imaging, the T_1_ of the gel was shortened by using gadolinium-containing contrast-agent (2.8 × 10^−3^ mL contrast-agent/mL water; Gadobutrol, Gadovist 1.0 mmol/mL, Bayer Schering Pharma, Newbury, UK). The complete cast was placed in a custom-made PVC container to which the gadolinium-gelatine solution was added, while avoiding the formation of air bubbles by slowly pouring the gadolinium-gelatine solution. The container was left overnight at 5 degrees Celsius for the gelatine to solidify.

#### 9.4T-MRI

From one cast (female, 57 years) a small cylindrical section with approximate dimensions of 7.5 mm radius and 15 mm height was cut with a diagonal pliers aided by an operating microscope (Universal S3, Carl Zeiss Meditec Inc., Dublin, California, USA, magnification 10–40×). This section was placed in a custom-made polymethyl methacrylate (Perspex) container and the above-mentioned gadolinium-gelatine solution was added. The Perspex container was subsequently placed in a desiccator for 10 minutes, followed by an ultrasonic cleaner for 10 minutes to remove air from the solution. During these steps, solidification of the solution was prevented by placing the container in a hot tap water bath. The container was left overnight at 5 degrees Celsius.

### Scan protocol

#### 7T-MRI

Imaging was performed on a 7T whole body system (Philips Healthcare, Cleveland, OH, USA) with a 60 cm bore diameter and maximum gradient strength/slew rate of 40 mT/m/200 mT/m/s, a custom-made 16-channel surface coil (MR Coils B.V. Zaltbommel, The Netherlands) for signal reception, and a volume transmit/receive coil for transmission (Nova Medical, Wilmington, MA, USA). The following imaging parameters were used for T1w imaging of both casts: TR 35 ms, TE 15 ms, flip angle 60 degrees, number of slices 300, bandwidth 106.4 Hz/pixel, TFE factor (number of excitations in each shot) 1004. The scanner resonance frequency was measured and adjusted between each shot (of approximately 35 s duration), to mitigate potential artefacts caused by frequency drift of the scanner. The field-of-view (FOV) was 150 × 150 × 30 mm^3^ with an acquired voxel size of 0.1 × 0.1 × 0.1 mm^3^. The scan duration was 3 h46 min.

#### 9.4T-MRI

Imaging was performed on a 9.4T/21 cm MR system (Varian Inc., Palo Alto, CA, USA) equipped with a gradient insert of 6 cm internal diameter with gradients up to 1T/m. A home-built inductively coupled 3 turn surface coil with an internal diameter of 21 mm was used that tightly fitted the sample. A 3D balanced SSFP sequence was used with the following scan parameters: TR/TE 8/4 ms, flip angle 45°, acquired voxel size of 0.03 × 0.03 × 0.03 mm^3^ and a FOV of 19.8 × 19.8 × 19.8 mm^3^, phase increments of 0°, 90°,180° and 270° for subsequent image acquisitions and 18 averages per phase increment. The acquisition time for a single 3D image (without phase increments) was about 58 minutes, resulting in a total acquisition time of 18 × 4 × 58 minutes = 70 hrs. The raw data were multiplied with a 3D Hanning window (660 points in every direction) and zerofilled to 1320 points in every direction before Fourier transformation. After Fourier transformation the complex images of the different phase increments were added and the absolute of the summed result was used for further quantification.

### Post-processing

#### Normalization

**Figure 3:**
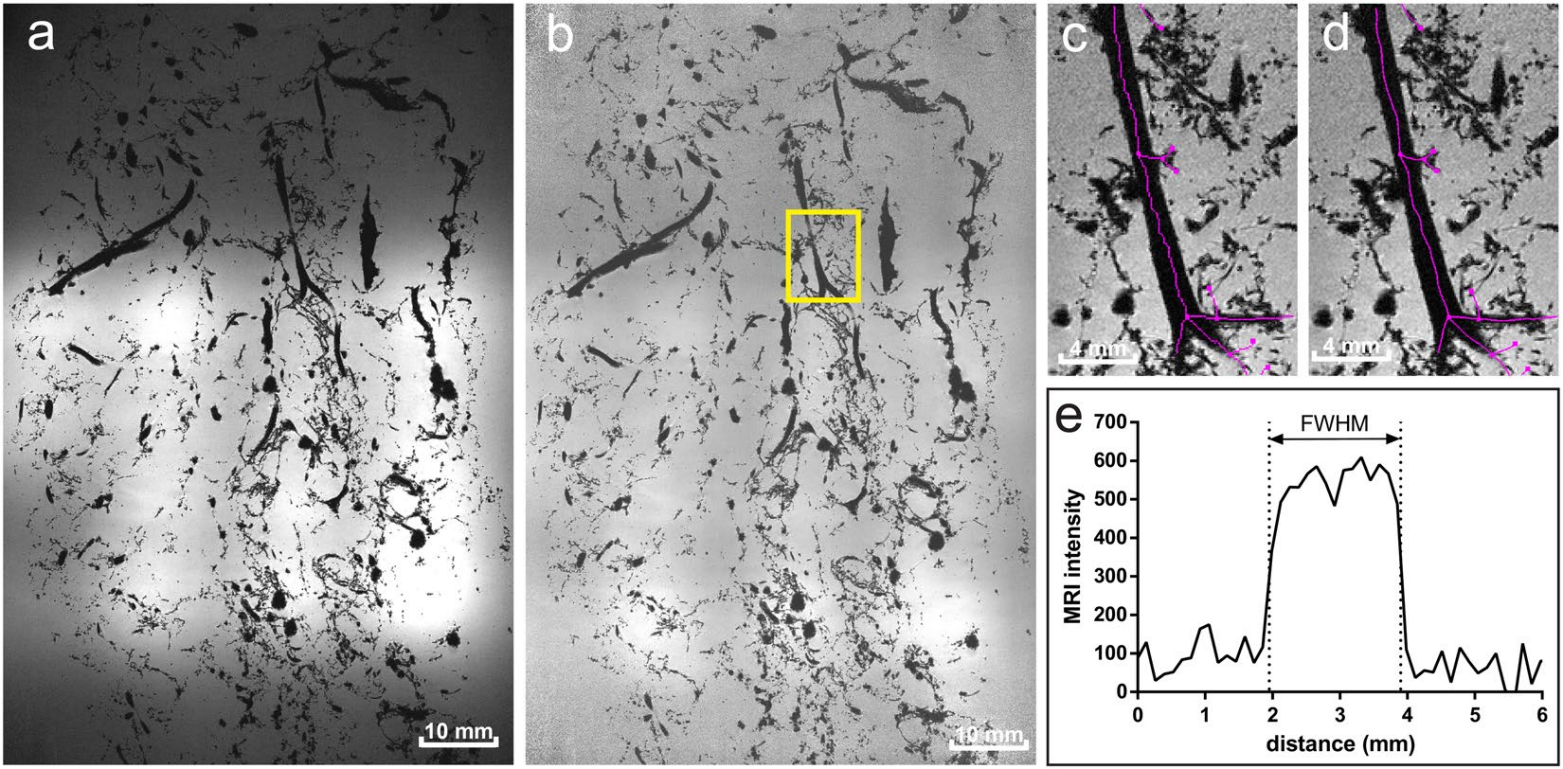
Post-processing steps. (**a**) Original 7T MRI single image showing the signal inhomogeneity due to local small receive coils. (**b**) Normalized image with yellow square marking close-up region of interest. (**c**) Close-up of non-smoothed centerlines. (**d**) Close-up of smoothed centerlines. (**e**) Example of one intensity profile trough the artery on which the estimated radius (obtained with full-width at half-maximum method) of the artery is depicted.

The small receiver elements of the 7T-MRI receiver coil caused signal fall-off at further distance from the coils, which hampered automated image analysis. This image inhomogeneity was corrected by data normalization. The density of the cast in the gelatine-gel was relatively low, thus justifying to ignore the presence of the cast in the gel, and to use a straightforward moving average filter (empirically chosen size 24 × 24 × 5) to obtain the normalization factor map for correction (Fig. 3a,b).

#### Centrelines

The 3D morphology of the cerebral arterial tree was described by artery centrelines and radii. Full automatic computation of centrelines was impossible due to many small (smaller than the voxel size) intertwined arteries that appeared as blobs. Hence, we used a semi-automatic approach aided by Simple Neurite Tracer (SNT, Version 2.0.2)^16^ in Fiji (ImageJ 1.50b, National Institutes of Health, USA)^17,18^. By selecting two locations in a vessel, SNT automatically generates the centreline between these two locations, based on voxel intensity values. This curved centreline is composed of many short, straight line pieces between automatically generated nodes. Bifurcations were made by starting a second path on a previously generated path Image contrast was inverted prior to generation of the centrelines in SNT (Fig. 3c).

Centrelines generated by SNT had a slight saw tooth profile, which was corrected by applying a smoothing algorithm (SmoothN, version 1.37) in MatLab (version 8.3, MathWorks Inc., Natick, MA, USA)^19,20^. On visual inspection a smoothing parameter of 20 proved to be optimal (Fig. 3d).

#### Radius calculation

Radius calculation was performed using a custom MatLab code that calculated the radius for all nodes. For each node, fifty radial intensity profiles were sampled by rotating the profile (in steps of 7.2 degrees) around the centreline at three locations spread over the straight line piece connecting the node of interest and its previous connecting node. We opted for using these three locations as we wanted each node to represent the average radius of the straight line connecting with the previous node.

SNT automatically generates the At locations where multiple arteries are very close, some of the radial intensity profiles will yield an overestimation of the actual radius as the second artery might also be included in radius calculation. As the radii obtained from the 50 radial intensity profiles should represent a circular artery, the circularity index (*Q*) was computed at each location to detect this overestimation:

$$Q=\frac{(4\,\times \pi \times A)}{{L}^{2}}$$

in which *A* is the cross-sectional area and *L* the circumferential length. In case of a perfect circle, Q is equal to 1, whereas in a shape less like a circle (*e*.*g*. an ellipse) Q decreases.

For each location, the fraction 1-Q largest radii were excluded. For example a Q of 0.80 would mean that the largest 20% of calculated radii were excluded. Visual inspection at a number of locations showed that this heuristic approach was effective at removing incorrect data profiles. The remaining radii for all points were averaged. After calculation the radii for all nodes, the results were saved in a file containing the arterial tree with all connectivity information and radii in a standard text file format (SWC format), as specified by Cannon *et al*.^21^.

### Validation of MRI measurements

The 7T-MRI measurements were validated by measuring the artery diameters at 50 locations by hand using a digital calliper on the cast (Mitutoyo Corp. Takatsu-ku, Japan, Accuracy ± 0.001 mm) aided by an operating microscope (Universal S3, Carl Zeiss Meditec Inc., Dublin, California, USA, magnification 10–40×) at the same location as in the MRI data using the calculated radii.

As the small arteries scanned with 9.4T-MRI could not be measured manually using a calliper, validation of 9.4T-MRI data was performed by scanning the container with the small cast section also on the 7T MRI, and comparing the measured radii at 50 arterial segments. Only segments with a 7T-MRI radius larger than one voxel (0.1 mm) were regarded valid for the validation measurements Therefore, segments with a smaller radius were excluded from analysis.

### Statistical analysis

Linear regression analysis was performed for the manual and 7T-MRI measurements, and for the 7T and 9.4T-MRI measurements. An intraclass correlation coefficient (ICC) using a Two-Way Mixed effect looking for absolute agreement of data and Bland-Altman plots with a range of agreement defined as a mean bias of ±2 standard deviations (SD) were performed. An acceptable threshold of bias was set at +/− 0.05 mm as this is expected to be comparable to variability in manual measurements. Linear regression analysis was used on the Bland-Altman plots to check for proportional differences. Linear regression and production of graphs was done using GraphPad Prism (version 6.01, GraphPad Software, Inc. La Jolla, CA, USA). All other statistical analyses were performed using IBM SPSS Statistics (version 23.0.0.0, IBM Company, Armonk, NY, USA). The datasets generated during and/or analysed during the current study and MatLab scripts are available from the corresponding author on reasonable request.

## Results

### 3D reconstruction results

**Figure 4:**
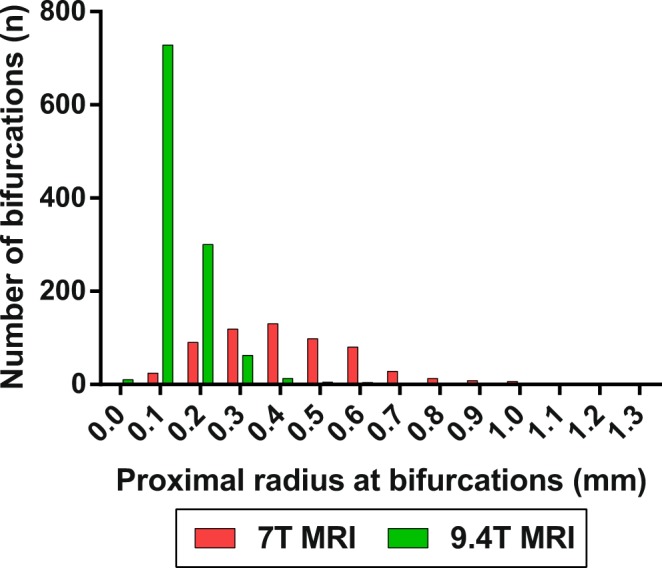
Absolute number of bifurcations for radius (mm) of proximal arteries of all bifurcations as measured on cast. Zero bin contains all values < 0.05 mm.

Figures 1 and 2 show photographs of the casts (Figs 1a and 2a), an example of the 7T and 9.4T-MRI data (Figs 2b, 3a,b) and the subsequent reconstructed arterial tree (Figs 2d and 3d). Using 7T and 9.4T-MRI arteries with radii down to 100 and 30 micrometres, respectively, could be reconstructed. The median radius for 7T-MRI was 0.4 mm (interquartile range 0.28–0.52 mm) and 0.12 mm (interquartile range 0.09–0.17 mm) for 9.4T-MRI (Fig. 4).

### MRI validation

**Figure 5:**
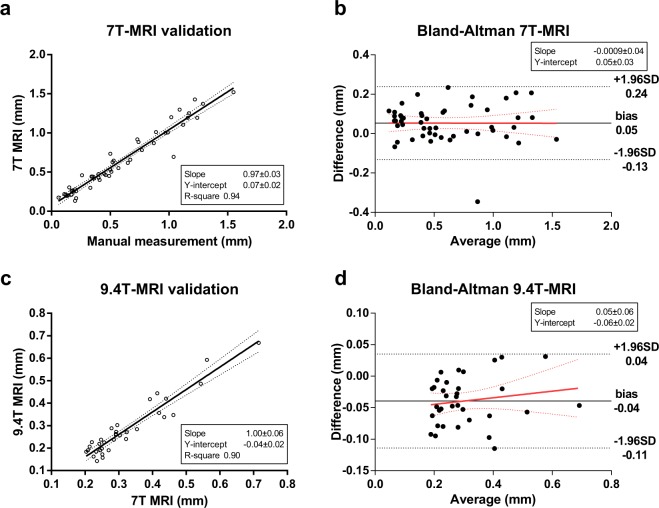
7T-MRI and 9.4T-MRI validation. (**a–b**) 7T-MRI validation by comparison of direct manual diameter measurements on cast using digital callipers and measurement performed on 3D reconstructed cast. (**c–d**) 9.4T-MRI validation by comparison of diameter measurements performed using 7T and 9.4T-MRI on the same segments in a casts. (**a**) Linear regression (solid line) analysis with 95%-confidence interval (dotted lines) of manual versus 7T MRI measurements. (**b**) Blant-altman plot of difference versus average for 7T MRI validation (difference: 7T MRI minus manual measurements). (**c**) Linear regression (solid line) analysis with 95%-confidence interval (dotted lines) of 7T versus 9.4T MRI. (**d**) Blant-Altman plot of difference versus average for 9.4T MRI validation (difference: 9.4T diameter minus 7T diameter).

Linear regression analysis of the manual measurements vs. the 7T MRI radii (Fig. 5a) showed a slope of 0.97 (95%-CI 0.90–1.04, Y-intercept: 0.07, R-squared = 0.94). The ICC of single measurements was 0.96 (95%-CI 0.91–0.98). The Bland-Altman plot showed a bias of −0.05 mm with limits of agreement between 0.23 and −0.13 mm (Fig. 5b). Linear regression analysis on the Bland-Altman showed no significant slope (−0.001 mm/mm, 95%-CI −0.072–0.070 mm/mm, p = 0.98).

Of the 50 segments selected for 9.4T-MRI validation, 16 had a 7T-MRI diameter <0.1 mm and were excluded, yielding 34 measurements for comparison with 7T-MRI. Linear regression analysis (Fig. 5c) showed a slope of 1.00 (95%-CI: 0.88–1.12, Y-intercept: −0.04, R-square = 0.90).The ICC of single measurements was 0.90 (95%-CI 0.43–0.97). The Bland-Altman plot showed a bias of 0.05 mm with limits of agreement between 0.04 and −0.11 mm (see Fig. 5d). Linear regression on the Bland-Altman showed no significant slope (0.052 mm/mm 95%-CI 0.062–0.17 mm/mm, p = 0.36).

## Discussion

This study shows that it is feasible to scan human cerebral arterial polymer casts with a human whole body 7T-MRI, providing the potential to characterize morphology of the human cerebral arterial tree over a large volume for vessels ≥0.1 mm in radius. Vessels down to a radius of 30 µm can be assessed at 9.4T-MRI (small bore system) showing the advantage of an increased resolution at the expense of a smaller sample volume. 7T-MRI performed well compared to manual measurements and 9.4T-MRI showed a very close agreement with the 7T-MRI measurements.

This technique could be an alternative to current techniques such as Micro-CT. Micro-CT will achieve a resolution slightly higher than that of the current presented 7T-MRI technique with a comparable scanning times of 2–6 hours^13,14^. The decision which technique is most feasible will depend on local availability and experience with either Micro-CT, 7T-MRI and 9.4T-MRI techniques. MRI has the advantage that it also allows for complementary *in-vivo* studies of the vasculature and blood flow^22,23^. However, *in vivo* CTA, MRI and 3D angiography can only be used to characterize the morphology of the larger end of the cerebral arterial tree, which makes it of limited use for the current purpose of characterizing the vascular tree in the range of 0.2–1 mm^6,7^.

The use of casts embedded in gadolinium-containing gelatine gel resulted in a high contrast between plastic arteries and surrounding gel and a high signal-to-noise ratio, even at the ultra-high resolutions achieved with 9.4T-MRI. This resulted in a new method for resolving the cerebral arterial tree at high resolutions.

At these high resolutions, the removal of air bubbles from the gadolinium-containing gelatine gel proved to be crucial. At the resolutions of 7T-MRI the artefacts caused by air could be avoided as long as the gel was carefully added into the PCV container such that no significant air bubbles were created. However, at the higher resolution used at 9.4T-MRI, sensitivity to artefacts caused by air increased and removal of even microscopic air bubbles became vital. This was successfully done by using a desiccator and an ultrasonic cleaner.

Because of the complex nature of the casts, fully automatic 3D reconstruction of the complete arterial network proved to be practically impossible. Hence, the current work opted for a semi-automatic approach. This approach is more time-consuming than an automatic approach but is still faster than traditional manual measurements on casts or dissected arterial trees. Using the current method, measuring a large number of assessable segments lengths and radii of a single cast could be captured in a 3–4 days (excluding MRI scan time). From our own experience capturing a comparable amount of data or radii using manual measurements on a single cast takes 1 week. If lengths are also required manual measurement on casts is not feasible and dissection is the preferred traditional method. Acquisition of a comparable amount of data on radii and lengths of arteries up to 300 micrometres from our own experience can take up to 1,5 months of full time work when done accurately.

In future research this method could be used to acquire large data-sets with quantitative knowledge and general descriptive of the morphology of the cerebral arterial tree. These could in turn be used for different purposes. One example would be generation of a general hemodynamic model for study on specific diseases, such as stroke and Moyamoya disease. A secondary option is to generate boundary conditions in patient-specific aneurysm flow models, for planning and evaluating the risk of by-pass surgery. The cerebral arterial resistance is known to be generated largely by the arterioles and capillaries^2,3^. Morphological data on the more distal arteries and arterioles in the cerebral arterial tree captured using 7T-MRI and 9.4T-MRI coupled with already existing morphological data on the larger cerebral arteries and the capillary network could prove valuable in estimating correct boundary conditions by enabling calculation of this resistance distally from the major cerebral arteries^6,19,20,24^.

The techniques used in the current study have their limitations. (a) Because of the limits to resolution, small side branches might be missed. The cerebral circulation is known to have many small perforating side branches which are often smaller than 10% in diameter of their parent artery^25,26^. For an artery smaller than two millimetres in diameter such a side branch would already be smaller than what is possible to measure using 7T-MRI at a resolution of 0.1 mm. (b) The used echo time might result in an increase in diameter appearance on MRI data. However, the current study found a good correspondence between manual measurements and MRI measurements which suggests that this effect was limited. (c) Intertwined arteries can appear larger in diameter when using purely a FWHM method. The circularity filter that was applied appeared to effectively avoid these errors, given the results of the validation. (d) An increasing field strength and resolution of 9.4T-MRI compared to 7T-MRI resulted in a decreased volume which could be captured, comparable to techniques such as micro-CT and confocal laser microscopy. This would limit acquisition of data on morphology of arteries smaller than 0.1 mm in radius of the cerebral arterial tree to only a small section of a cast. When acquiring general descriptive morphological data on the complete cerebral arterial tree, such a small section might theoretically not be representative for the remainder of the cerebral arterial tree. However, the use of multiple small sections of the same cast could give a better representation of the cerebral arterial tree. (e) Finally, the quality of the casts which are used might influence the acquired morphology and its representation of the actual physiological situation. The radius of the arteries in the casts will depend on the pressure used during injection and shrinkage of the injection material during solidification and erosion. Pressure can be controlled and kept at a physiological level during production of casts. Shrinkage of Araldite F as used in the current study is known to be 3.17 ± 0.52% and thus minimal^15^. Gravity may result in deformation off the casts, especially after air-draying the casts. We expect that this doesn’t affect radius and length measurements but does have its effects when morphological branching angles are of interest.

In conclusion, 7T and 9.4T-MRI scanning of plastic casts embedded in gadolinium-containing gelatine gel makes it feasible to obtain quantitative characterization of the cerebral arterial morphology in a timely manner, easing characterisation of multiple casts. This data might help in modelling and understanding various cerebrovascular diseases.

## References

[1] Hirsch S, Reichold J, Schneider M, Székely G, Weber B (2012). Topology and hemodynamics of the cortical cerebrovascular system. J. Cereb. Blood Flow Metab..

[2] Baumbach GL, Heistad DD (1985). Regional, segmental, and temporal heterogeneity of cerebral vascular autoregulation. Ann. Biomed. Eng..

[3] Hall CN (2014). Capillary pericytes regulate cerebral blood flow in health and disease. Nature.

[4] Cassot F, Lauwers F, Fouard C, Prohaska S, Lauwers-Cances V (2006). A novel three-dimensional computer-assisted method for a quantitative study of microvascular networks of the human cerebral cortex. Microcirculation.

[5] Cassot F (2010). Branching patterns for arterioles and venules of the human cerebral cortex. Brain Res..

[6] Rossitti S, Löfgren J (1993). Vascular dimensions of the cerebral arteries follow the principle of minimum work. Stroke.

[7] Lescher S, Samaan T, Berkefeld J (2014). Evaluation of the pontine perforators of the basilar artery using digital subtraction angiography in high resolution and 3D rotation technique. AJNR. Am. J. Neuroradiol..

[8] Horsfield K, Woldenberg MJ (1989). Diameters and cross-sectional areas of branches in the human pulmonary arterial tree. Anat. Rec..

[9] Mittal N (2005). A computer reconstruction of the entire coronary arterial tree based on detailed morphometric data. Ann. Biomed. Eng..

[10] Koike K, Ohnuki T, Ohkuda K, Nitta S, Nakada T (1986). Branching architecture of canine pulmonary arteries: a quantitative cast study. Tohoku J. Exp. Med..

[11] Nordsletten DA, Blackett S, Bentley MD, Ritman EL, Smith NP (2006). Structural morphology of renal vasculature. Am. J. Physiol. Heart Circ. Physiol..

[12] Du Plessis A, Rossouw P (2015). X-ray computed tomography of a titanium aerospace investment casting. Case Stud. Nondestruct. Test. Eval..

[13] du Plessis A, le Roux SG, Guelpa A (2016). Comparison of medical and industrial X-ray computed tomography for non-destructive testing. Case Stud. Nondestruct. Test. Eval..

[14] Dalstra, M. *et al*. Hard X-ray micro-tomography of a human head post-mortem as a gold standard to compare X-ray modalities. *Soc*. *Photo-Optical Instrum*. *Eng*. *Conf*. *Ser*. **9967** (2016).

[15] Van Der Zwan A, Hillen B (1990). Araldite F as injection material for quantitative morphology of cerebral vascularization. Anat. Rec..

[16] Longair MH, Baker DA, Armstrong JD (2011). Simple Neurite Tracer: open source software for reconstruction, visualization and analysis of neuronal processes. Bioinformatics.

[17] Schindelin J (2012). Fiji: an open-source platform for biological-image analysis. Nat. Methods.

[18] Schneider CA, Rasband WS, Eliceiri KW (2012). NIH Image to ImageJ: 25 years of image analysis. Nat. Methods.

[19] Garcia D (2010). Robust smoothing of gridded data in one and higher dimensions with missing values. Comput. Stat. Data Anal..

[20] Garcia D (2011). A fast all-in-one method for automated post-processing of PIVdata. Exp. Fluids.

[21] Cannon RC, Turner DA, Pyapali GK, Wheal HV (1998). An on-line archive of reconstructed hippocampal neurons. J. Neurosci. Methods.

[22] van Ooij P (2013). Quantification and visualization of flow in the Circle of Willis: Time-resolved three-dimensional phase contrast MRI at 7 T compared with 3 T. Magn. Reson. Med..

[23] Bouvy WH (2016). Assessment of blood flow velocity and pulsatility in cerebral perforating arteries with 7-T quantitative flow MRI. NMR Biomed..

[24] Cassot F, Lauwers F, Lorthois S, Puwanarajah P, Duvernoy H (2009). Scaling laws for branching vessels of human cerebral cortex. Microcirculation.

[25] Marinkovic SV, Milisavljevic MM, Kovacevic MS, Stevic ZD (1985). Perforating branches of the middle cerebral artery. Microanatomy and clinical significance of their intracerebral segments. Stroke.

[26] Umansky F (1985). The perforating branches of the middle cerebral artery. A microanatomical study. J. Neurosurg..

